# Emerging Mechanisms of Abdominal Aortic Aneurysm

**DOI:** 10.1007/s11883-026-01392-5

**Published:** 2026-01-30

**Authors:** Botao Zhu, Y. Eugene Chen, Yanhong Guo

**Affiliations:** 1 Department of Internal Medicine, Frankel Cardiovascular Center, University of Michigan, Ann Arbor, MI USA; 2 Department of Cardiovascular Medicine, The Second Xiangya Hospital of Central South University, Changsha, Hunan China

**Keywords:** Abdominal aortic aneurysm, Pathogenesis, Inflammation, Therapeutic

## Abstract

**Purpose of Review:**

Abdominal aortic aneurysm (AAA) is a progressive and often fatal vascular disease for which effective pharmacological therapies are lacking. This review synthesizes recent mechanistic advances in AAA pathogenesis and evaluates their translational significance for therapeutic development.

**Recent Findings:**

Single-cell and spatial transcriptomic findings have delineated marked cellular heterogeneity within aneurysmal tissue, revealing dynamic interactions among vascular and immune cell populations. Vascular smooth muscle cell phenotypic modulation and programmed cell death compromise aortic wall integrity, while endothelial dysfunction promotes leukocyte recruitment and mediates early vascular responses. Infiltrating macrophages, neutrophils, and adaptive immune cells orchestrate chronic inflammation and extracellular matrix degeneration, whereas eosinophils and regulatory T cells exert context-dependent protective effects. Local factors, including intraluminal thrombus and perivascular adipose tissue, as well as systemic modulators such as dyslipidemia, gut microbiota, and sex hormones, further shape disease initiation and progression. These mechanistic insights have identified novel therapeutic targets, including inhibitors of regulated cell death, immunomodulatory agents, lipid-lowering interventions, and microbiome-directed strategies, and potential biomarkers for earlier diagnosis and improved risk stratification.

**Summary:**

Emerging mechanistic insights have highlighted the complex interplay among vascular cells, immune cells, the local microenvironment, and systemic modulators in the pathogenesis of AAA. Integrating mechanistic insights with translational research will be crucial in developing targeted interventions that pave the way for effective AAA therapies.

**Graphical Abstract:**

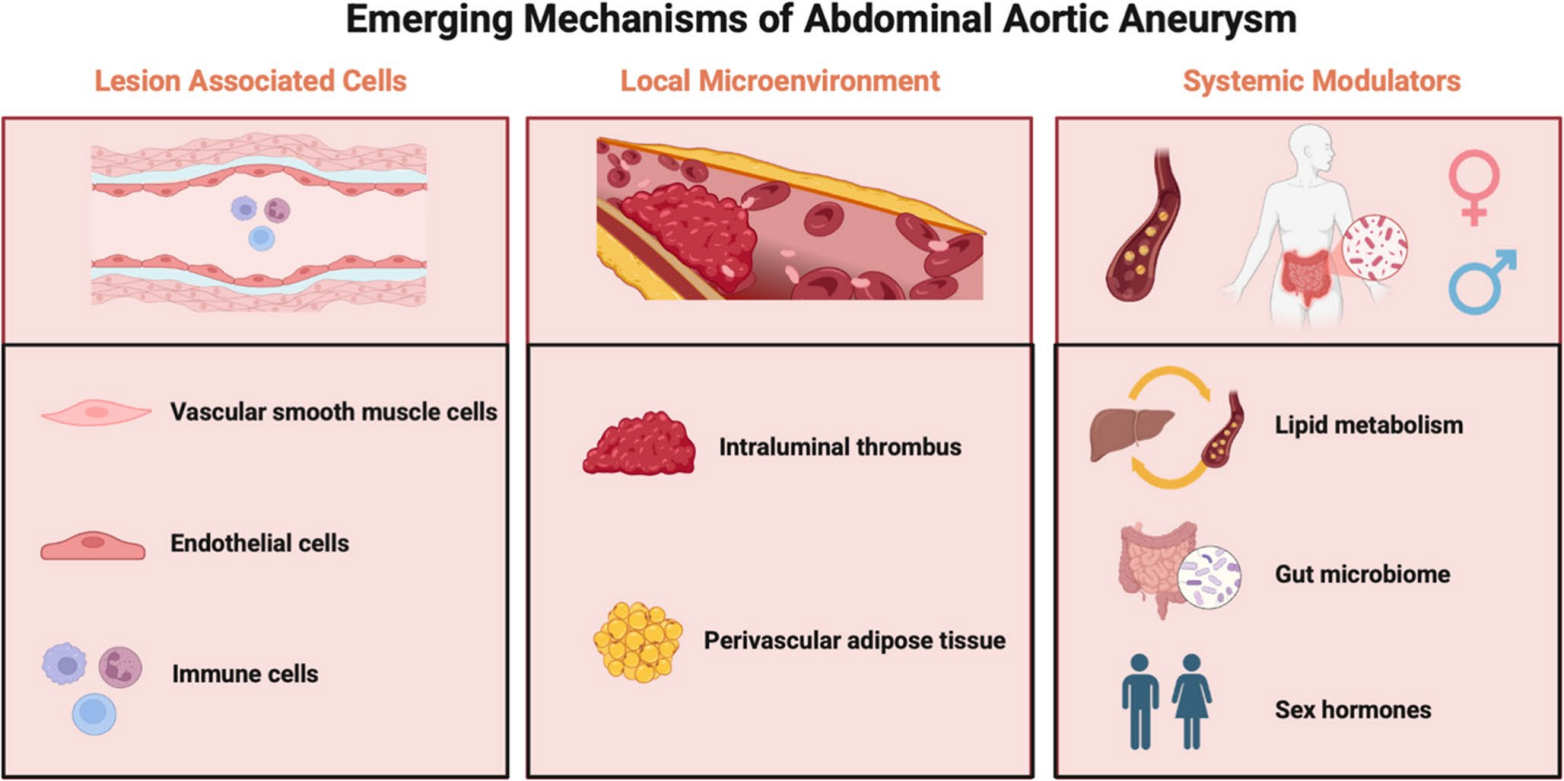

## Introduction

Abdominal aortic aneurysm (AAA) is characterized by a progressive, localized dilation of the abdominal aorta. Most AAAs remain clinically silent until rupture, which precipitates catastrophic hemorrhage and carries a mortality rate exceeding 80% [1, 2]. Globally, aortic rupture is estimated to cause 150,000–200,000 deaths annually, a figure likely underestimated given the asymptomatic nature of the disease [1, 3]. For AAA at a high rupture risk, open surgical repair or endovascular aneurysm repair (EVAR) represents the standard of care [4, 5]. However, both procedures are associated with considerable costs, perioperative risks, and the potential for recurrence. In patients with smaller aneurysms or those deemed unsuitable for surgery, no pharmacological therapy has yet been proven to attenuate disease progression, leaving a critical therapeutic gap [4]. Current management strategies primarily focus on preventing rupture by addressing risk factors associated with AAA. Established risk factors include advanced age, smoking, male sex, family history, hypertension, and hyperlipidemia [5]. Although lifestyle modifications such as smoking cessation may reduce the risk of disease onset, patients with established AAA urgently require therapeutic strategies that directly and effectively target the underlying pathological mechanisms. Consequently, elucidating the molecular and cellular mechanisms driving AAA pathogenesis is essential for identifying novel therapeutic targets and advancing mechanism-based interventions.

**Fig. 1:**
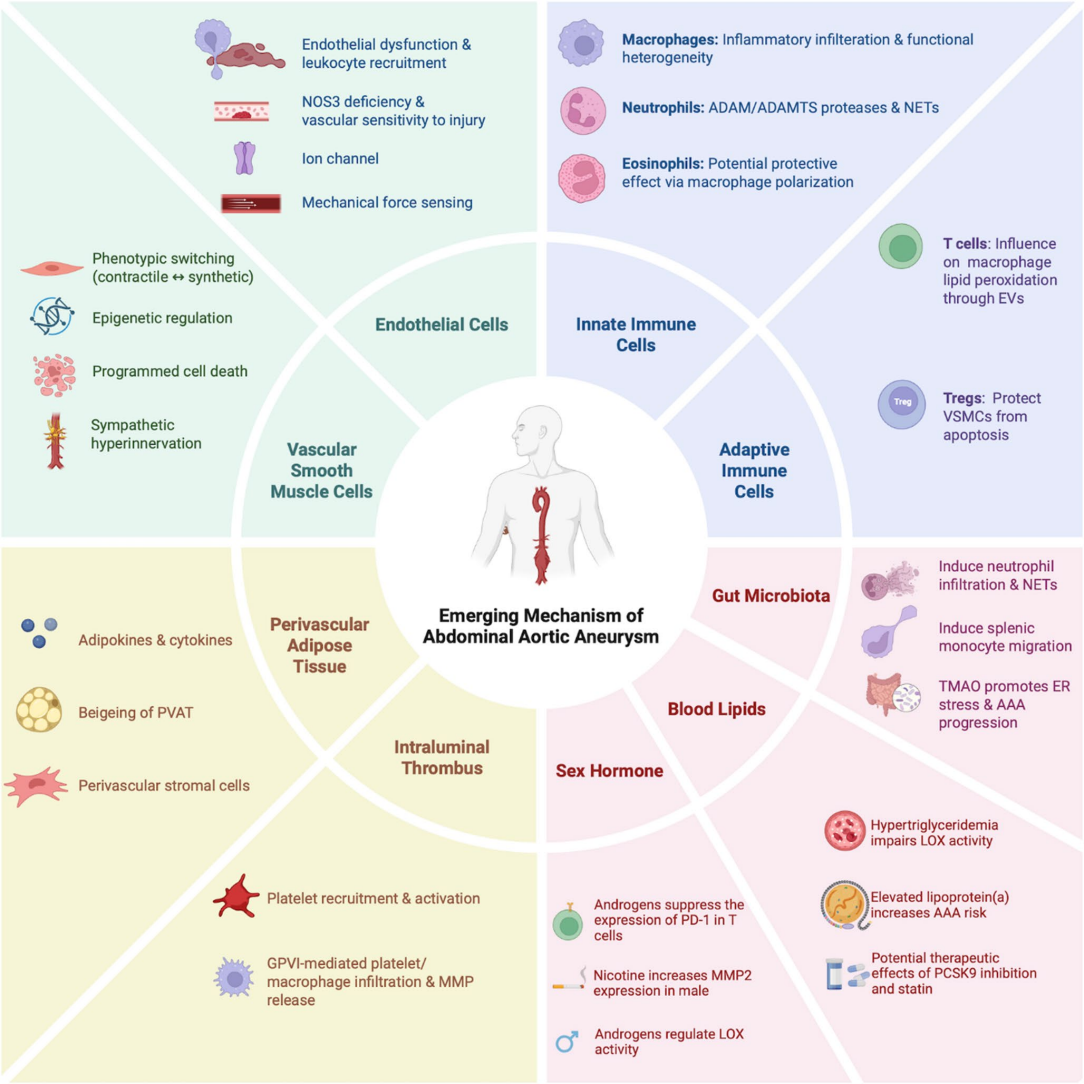
Emerging mechanisms involved in abdominal aortic aneurysm (AAA). Recent studies have revealed that multiple cellular and molecular pathways contribute to the development and progression of AAA. Vascular smooth muscle cells (VSMCs) undergo phenotypic switching, epigenetic regulation, programmed cell death, and sympathetic hyperinnervation. Endothelial cells exhibit dysfunction, endothelial nitric oxide synthase (NOS3) deficiency, abnormal ion channel activity, and altered mechanical force sensing, facilitating leukocyte recruitment to the aortic wall. Innate immune cells contribute via macrophage heterogeneity, neutrophil extracellular traps (NETs), and eosinophil-mediated macrophage polarization. Adaptive immune cells, including T cells and regulatory T cells (Tregs), regulate vascular inflammation and VSMC apoptosis. Within the local vascular microenvironment, perivascular adipose tissue (PVAT) regulates vascular homeostasis through its stromal cells, adipose tissue beiging and the release of various adipokines and cytokines. Intraluminal thrombus promotes AAA by platelet recruitment, glycoprotein VI (GPVI) -mediated platelet/macrophage infiltration, and matrix metalloproteinase (MMP) release. Systemic factors, including gut microbiota, dysregulated lipid metabolism, and sex hormones, further drive AAA progression. Sex hormones, especially androgens, contribute to sex dimorphism in AAA by inhibiting programmed cell death protein-1 (PD-1) expression in T cells, increasing MMP2 expression, and modulating lysyl oxidase (LOX) activity. Gut microbiota modulates AAA through neutrophil infiltration, splenic monocyte migration, and TMAO-induced ER stress. The role of abnormal lipid levels in AAA has also gained increasing attention. Lipid metabolism contributes to AAA pathogenesis through hypertriglyceridemia and elevated lipoprotein(a), while therapeutic targets such as PCSK9 inhibitors and statins have shown potential value in AAA treatment. Together, these mechanisms highlight the complex pathogenesis of AAA and provide potential therapeutic opportunities

In recent years, the advent of high-resolution technologies, such as single-cell RNA sequencing (scRNA-seq) and spatial transcriptomics, has provided unprecedented opportunities to explore the dynamic changes in cellular phenotypes and subpopulations in AAA [6–9]. Emerging evidence indicates that AAA progression is orchestrated by dynamic interactions among vascular smooth muscle cells (VSMCs), endothelial cells (ECs), and diverse immune cell populations, each contributing distinct yet interconnected pathological processes. VSMC phenotypic switching and death weaken the aortic wall, while EC dysfunction promotes leukocyte recruitment, intramural inflammation, and altered vascular permeability. Infiltrating immune cells, including macrophages, T cells, and neutrophils, amplify inflammatory cascades, proteolytic degradation, and cellular injury. These cellular events converge with intraluminal thrombus (ILT) and perivascular adipose tissue (PVAT) and systemic modulators such as blood lipids, gut microbiota, and sex hormones to drive aneurysm expansion (Fig. 1). In this review, we summarize the emerging mechanisms of each component in the initiation and progression of AAA, and discuss potential targets for therapeutic intervention, aiming to provide insights for the development of new treatment strategies for AAA.

## VSMC: Central Structural Cells in AAA

VSMCs are the primary cells of the aortic media. For a long time, VSMC dysfunction and apoptosis have been considered the key pathological drivers of arterial wall structural integrity destruction, leading to AAA expansion and rupture [10].VSMCs are a dynamic cell population with unique plasticity, allowing them to switch between contractile and synthetic phenotypes [11]. In healthy vessels, contractile VSMCs highly express contractile function proteins to maintain normal arterial tension. Under stress conditions, VSMCs exhibit a proliferative and inflammatory synthetic phenotype. Additionally, during the progression of AAA, VSMCs are lost through various forms of cell death, such as apoptosis, pyroptosis, and ferroptosis [12–14]. Here, we focus on recent studies elucidating the pivotal roles of VSMCs in AAA pathogenesis.

### Epigenetic Regulation of VSMC Phenotype

Accumulating evidence highlights the critical role of epigenetic mechanisms in regulating VSMC phenotype and function during AAA development. Chakraborty et al. found that interferon regulatory factor 3 (IRF3) suppresses the expression of SMC contractile genes by inducing chromatin remodeling [6]. Mechanistically, IRF3 interacts with the chromatin modifier EZH2, which drives the recruitment of H3K27me3 to the promoters of SMC-specific contractile genes such as *Acta2* and *Mylk*, thereby silencing their transcription. In addition, cytosolic DNA sensing through the STING pathway has been identified as a critical epigenetic driver of SMC phenotypic switching. Our previous research also elucidates that phenotypic switching and apoptosis in VSMCs are regulated by chromatin remodeling [15]. BAF60c, a subunit of the SWI/SNF chromatin-remodeling complex, plays a key role in epigenetic regulation by altering nucleosome positioning and chromatin accessibility. On the one hand, BAF60c acts as a co-activator of SRF, maintaining the contractile phenotype of VSMCs. On the other hand, it functions as an inhibitor of NF-κB/P65 to alleviate vascular inflammation. Specific knockout of *Baf60c* in VSMCs significantly exacerbates Ang II and porcine pancreatic elastase (PPE)-induced AAA formation in mice, accompanied by enhanced SMC phenotypic switching and apoptosis, elastic fiber degradation, and inflammatory cell infiltration [15]. Knocking down adenosine kinase in VSMCs reduced TNF-α-induced expression of key inflammatory and AAA-related genes by approximately 50%, including IL1B, IL6, VEGFA, and MMP3, through an epigenetic mechanism independent of adenosine receptors [16]. Although these findings highlight the epigenetic plasticity underlying VSMC phenotypic conversion, further research is needed to clarify the precise mechanisms of epigenetic regulation and to assess the feasibility of therapeutic interventions targeting these pathways in AAA.

### Sympathetic Regulation of Vascular Remodeling

Several studies have underscored the critical role of perivascular sympathetic nerves in regulating VSMC phenotype. Excessive sympathetic activation leads to elevated norepinephrine release, which has been shown to induce the proliferation of pulmonary artery smooth muscle cells, thereby contributing to pulmonary vascular remodeling [17]. Similarly, sympathetic hyperinnervation significantly promotes AAA development by facilitating VSMC phenotype switching via the extracellular ATP (eATP)/P2rx4/p38 signaling pathway [18]. Furthermore, scRNA-seq has revealed that sympathetic hyperinnervation is driven by Sema4D, a molecule secreted by osteoclast-like cells, which exerts its effect via binding to Plxnb1 receptor on sympathetic nerves [18].

### VSMC Death Pathways

In AAA, various distinct modes of VSMC death have been reported. Studies have demonstrated that both human VSMCs from AAA patients and cells from AAA murine models exhibit significantly reduced levels of ganglioside GM3 and decreased expression of its synthesizing enzyme, GM3 synthase (ST3GAL5) [19]. The downregulation of GM3 contributes to iron deposition, triggering ferroptosis in VSMC [19]. Gasdermin D (GSDMD), a known executor of pyroptosis, has also been investigated in the context of AAA. Interestingly, Gao and colleagues reported that GSDMD-induced pyroptosis was not observed in human aortic smooth muscle cells (HASMCs) during their examination of GSDMD function in AAA [20]. However, in an AngII-induced AAA model, upregulation of GSDMD in VSMCs promotes the expression of ornithine decarboxylase 1 (ODC1) through the endoplasmic reticulum stress-C/EBP homologous protein (CHOP) pathway [20]. Elevated levels of the end product putrescine, the metabolic product of ODC1 activity, were illustrated to modulate VSMC phenotype and inflammatory status, thereby contributing to AAA progression. Pharmacological inhibition of ODC1, such as with difluoromethylornithine, has emerged as a potential therapeutic approach for AAA, although further confirmation is required. Despite accumulating experimental evidence supporting a central role of programmed VSMC death in AAA pathogenesis, therapeutic development remains limited [14]. Therefore, in addition to identifying new molecular components involved in these pathways, future research should prioritize the discovery and refinement of selective inhibitors with minimal off-target effects.

## Endothelial Cells: Gatekeepers of Vascular Homeostasis

Endothelial dysfunction is increasingly recognized as a pivotal initiating factor in AAA pathogenesis. In hypercholesterolemic murine models, exposure to cigarette smoke exacerbates vascular inflammation and promotes aneurysm formation, primarily by enhancing monocyte recruitment through endothelial-derived colony-stimulating factor-1 (CSF-1). Notably, pharmacological inhibition of CSF-1 or its receptor (CSF-1R) significantly reduces aneurysm incidence [21], underscoring the pathological relevance of this axis in AAA development.

Beyond its role in inflammation, the endothelium plays a fundamental part in regulating vascular tone and leukocyte adhesion via nitric oxide (NO) production, which is mediated by endothelial nitric oxide synthase (NOS3) [21]. Endothelial NOS3 deficiency has been described to predispose to AAA formation. However, the susceptibility is pronounced only in the presence of vascular injury, such as cigarette smoke. These findings suggest that NOS3 loss sensitizes the vasculature to environmental injury, thereby contributing to the initiation of aneurysms. Recent studies have also implicated the endothelial ion channel pannexin-1 (Panx1) in AAA progression. Mechanistically, Panx1 facilitates neutrophil recruitment by mediating ATP release, activates macrophages through engagement of the P2 × 7 receptor, and modulates VSMC activation and vascular remodeling via P2Y2 signaling pathways [22]. These findings uncover a novel role for ion channels in orchestrating inflammatory and remodeling processes during AAA development. Additionally, endothelial cells possess mechanosensory capabilities that enable them to detect and transduce hemodynamic mechanical forces. Zhang et al. identified that the cytoskeletal scaffold protein angiomotin-like 2 (AmotL2) transmits mechanical signals from the plasma membrane to the nuclear envelope, and endothelial AmotL2 deficiency results in enhanced vascular inflammation and AAA formation [23].

Collectively, these findings emphasize the role of endothelial cells as dynamic ‘gatekeepers” of vascular homeostasis. Continuously sensing biochemical and mechanical stimuli within the vascular microenvironment, endothelial cells initiate extensive intercellular communication once pathogenic thresholds are exceeded or endothelial integrity is compromised, thereby driving the onset and progression of AAA.

## Immune Cells: Orchestrators of Chronic Inflammation and Tissue Degradation

### Macrophages Initiate and Sustain AAA Inflammation

A hallmark feature of AAA is the prominent infiltration and activation of inflammatory cells within the abdominal aortic wall, with monocytes and macrophages as the predominant cell populations involved in all stages of disease progression [24]. These immune cells influence AAA pathogenesis from initiation to structural degradation and even participate in the repair process.

Macrophages in AAA originate from two primary sources: circulating monocytes and resident aortic macrophages. While monocyte-derived macrophages are generally considered the principal contributors to inflammation and AAA expansion [25], the role of resident macrophages remains less defined and warrants further investigation [25, 26]. Recent scRNA-seq analysis of elastase-treated murine models revealed a sustained time-dependent increase in macrophage numbers within the aortic wall [8]. Functional clustering analyses further uncovered marked functional heterogeneity among macrophage subpopulations. Inflammatory macrophages derived from monocytes express high levels of pro-inflammatory mediators and proteases that promote extracellular matrix (ECM) degradation, such as matrix metalloproteinase 9 (MMP9) and cathepsins (CTSC, CTSD, or CTSS). In contrast, reparative macrophages, also derived from monocytes, were enriched for Arginase 1 and IL-10, indicating potential roles in tissue repair and immune regulation.

Two distinct subsets of resident macrophages have been identified: one enriched in cytokine production, and another exhibiting self-renewal potential. Notably, traditional M1/M2 polarization markers failed to distinctly classify these populations, as subsets co-expressed both pro- and anti-inflammatory genes, with differences primarily in expression intensity rather than in distinct polarization states [8]. These findings display a more nuanced, context-dependent macrophage landscape in AAA, challenging the utility of the M1/M2 paradigm.

While macrophages have long been considered key effectors of vascular inflammation and ECM breakdown in AAA, recent findings have expanded our understanding of macrophage functional diversity in AAA. For example, exposure to elastase in atherosclerotic mice promotes monocyte recruitment, linking AAA formation to existing vascular disease [21]. Within plaques, TREM2⁺ macrophages contributed to elastin degradation and weakening of the vascular wall [21]. In another research, TREM-1 expression has been localized to Ly6C Hi macrophages in AngII-induced models, where it amplifies inflammatory monocyte recruitment and local cytokine production [27]. Additionally, adenosine deaminase acting on RNA 1 (ADAR1), an RNA-editing enzyme previously linked to SMC phenotypic switching, has been implicated in macrophage-mediated AAA progression [28]. ADAR1 interacts with Drosha to drive vascular inflammation and aneurysm development [29], and its hematopoietic cell-specific deletion markedly attenuates aortic dilation and elastin degradation [29]. Paradoxically, MMP12, a macrophage-derived protein traditionally associated with ECM degradation and aneurysm pathology [30], was recently validated to exhibit a protective role. MMP12 deficiency promoted AAA formation by activating the complement and neutrophil extracellular trap (NET) formation [31]. This unexpected finding underscores the multifaceted and context-specific roles of macrophage-derived proteases in AAA.

Collectively, these studies support the concept that monocytes and macrophages participate in both early inflammatory signaling and later matrix degradation during AAA progression. Their subpopulation-specific roles depend heavily on the surrounding microenvironment, and future studies leveraging advanced imaging, single-cell analytics, and integrative bioinformatics analyses will be necessary to clarify their stage-specific contributions and therapeutic potential.

### Neutrophils Fuel Vascular Inflammation and Wall Degeneration

Neutrophils are abundantly present in the outer layers of the aneurysmal wall and the ILT, where they contribute to ECM degradation by releasing proteolytic enzymes [32]. A recent scRNA-seq study of human AAA tissues revealed the enrichment of metalloendopeptidase⁺ (MME^+^) neutrophils, which were largely absent in healthy aortas [9]. These neutrophils exhibited high expression of A Disintegrin and Metalloproteinase (ADAM) and A Disintegrin and Metalloproteinase with Thrombospondin Motifs (ADAMTS) family members [9], key regulators of ECM remodeling and inflammation. Functional analyses positioned MME⁺ neutrophils as pivotal hubs within immune cell communication networks, coordinating inflammatory responses during aneurysm progression [9]. Spatial transcriptomic mapping showed that these neutrophils were localized near CCL5⁺ macrophages [9], suggesting coordinated inflammatory signaling between these cell populations. Elucidating the interactions between neutrophils and other immune cells in AAA may provide novel therapeutic entry points to mitigate disease progression.

In addition to their enzymatic functions, neutrophils contribute to AAA through the release of neutrophil extracellular traps (NETs), which are large extracellular mesh-like structures composed of chromatin and granule proteins that trap pathogens [33]. Circulating markers of NETs are significantly elevated in AAA patients and correlate with disease severity [34]. Mechanistically, NETs can induce VSMC ferroptosis by inhibiting the PI3K/AKT signaling pathway [34]. Intriguingly, mesenchymal stem cell-derived extracellular vesicles (EVs) have been shown to reduce NET release in AngII-induced AAA, pointing toward a potential regenerative therapy [34]. Ibrahim et al. reported that NET targeting agents are most effective when acting within the ILT, limiting neutrophil activity and aneurysm progression in murine models [32], revealing that NETs may accumulate and exert their pathogenic functions within ILT. Moreover, inhibition of upstream NET formation yielded more pronounced benefits than downstream blockage, because neutralizing already-formed NETs is challenging [32].

### Potential Protective Role of Eosinophils

Eosinophils are innate immune cells capable of releasing various cationic proteins and have been clinically linked to a range of cardiovascular diseases, including atherosclerosis, myocardial infarction, and atrial fibrillation [35]. Eosinophil infiltration has been observed in both human and experimental AAA lesions, where they appear to exert protective effects by secreting IL-4 and cationic proteins such as mEar1 [36]. These mediators promote macrophage and monocyte polarization toward anti-inflammatory phenotypes and suppress NF-κB activation in vascular cells, thereby limiting aortic inflammation [36]. Consistently, Zhang et al. reported that group 2 innate lymphoid cells promote eosinophil differentiation, which in turn reduces VSMC apoptosis, suppresses endothelial adhesion molecule expression, and limits the polarization of pro-inflammatory Ly6Chi monocytes [37]. However, given the dual roles that eosinophils may play in other cardiovascular settings, including pro-atherogenic and pathogenic effects [38, 39], further studies are needed to delineate the precise functions of eosinophils and their mediators in AAA development and progression.

### Adaptive Immune Cells in AAA

Adaptive immune cells and their effector products also play a substantial role in AAA development. The detection of IgG autoantibodies, especially targeting normal aortic proteins, in AAA patients suggests an autoimmune component to disease pathogenesis [40]. In the elastase-induced AAA model, the proportion of T cells increases significantly, from 3.5% to 14.8% of immune cells, while B cells remain at relatively low levels (~ 2%) [8].

T cells influence AAA progression not only through cytokine production but also via metabolic regulation of other immune cells. In elastase-induced AAA models and in clinical AAA patients, infiltrating T cells upregulate pyruvate kinase muscle isozyme 2, which releases EVs containing higher levels of phospholipids and polyunsaturated fatty acids [41]. These EVs induce iron accumulation and lipid peroxidation in macrophages, thereby exacerbating vascular inflammation. Indeed, EVs derived from AAA patient plasma replicate these effects in vitro [41].

Regulatory T cells (Tregs) may provide a counter-regulatory mechanism. Li et al. found that Tregs are gradually recruited from the peripheral circulation during AAA progression in a PPE-induced model. Bulk RNA sequencing and single-cell T cell receptor profiling revealed that increased expression of Tff1, which protected against AAA by suppressing VSMC apoptosis through the ERK1/2 pathway [42]. These findings suggest AAA-specific antigens may exist, potentially enabling antigen-specific Treg-based therapeutic strategies.

## Local Microenvironment of the Aorta

The local vascular microenvironment, particularly ILT and PVAT, plays a critical role in modulating AAA progression. These structures not only respond to vascular injury but also actively participate in the inflammatory and remodeling processes during aneurysm pathogenesis.

### ILT: a Reservoir of Inflammatory and Proteolytic Activity

Approximately 70–80% of AAA patients develop non-occlusive ILT [44], which is driven by a combination of elastin degradation, altered hemodynamics, and platelet activation. These processes culminate in thrombin generation and the stabilization of the thrombus along the luminal surface of the aortic wall [43]. Transcriptomic analyses have shown that ILT is enriched with platelet-associated genes, including glycoprotein VI (GPVI), a key mediator of platelet adhesion and activation [44]. Functionally, GPVI deficiency in experimental models leads to reduced neutrophil and platelet infiltration, lowers circulating levels of MMPs and osteopontin (OPN), and preserves VSMC contractile phenotype and viability. These changes collectively limit aortic expansion and inflammation [45].

Additional studies reinforce the role of platelet-macrophage interactions in exacerbating vascular injury. Platelets facilitate macrophage infiltration and promote local OPN expression in the aortic wall, thereby enhancing ECM degradation, platelet adhesion, and ILT formation. Conversely, platelet depletion has been indicated to attenuate vascular inflammation and structural deterioration [46]. Furthermore, mechanically activated platelets also increase the expression and activity of platelet-derived MMPs, further driving ECM breakdown and vascular wall destabilization in AAA [47].

### PVAT: a Dynamic Modulator of Aortic Inflammation and Remodeling

The outermost layer of the aortic wall is composed of PVAT, which acts as a bidirectional signaling hub, sensing cues released from the vessel wall and transmitting signals inward to maintain vascular homeostasis [48, 49]. In the setting of AAA, PVAT becomes activated and undergoes phenotypic changes that contribute to disease progression [50–52]. Following vascular injury, adjacent PVAT undergoes beiging, characterized by increased thermogenic gene expression and altered secretory profiles. This transition leads to the release of neuregulin-4, which modulates local inflammation by promoting alternative macrophage activation and dampening pro-inflammatory signaling [50]. Beyond adipocytes that secrete adipokines, perivascular stromal cells (PVSCs) within PVAT also play indispensable roles. SM22α^+^ PVSCs maintain the balance between adipogenic and myofibrogenic differentiation through the PGC1α-YAP signaling axis. Loss of PGC1α disrupts this balance, skewing PVSCs toward a myofibroblast-like phenotype that accelerates AAA formation [53]. These mechanistic insights support the concept that PVAT is not merely a passive fat depot but an active participant in vascular inflammation and remodeling. Targeting PVAT-derived mediators or modulating PVSC differentiation could offer novel therapeutic strategies in treating AAA.

ILT and PVAT are critical components of the aortic microenvironment that actively shape the pathophysiological landscape of AAA. Continued exploration of these local compartments may uncover novel therapeutic targets and improve our understanding of AAA progression.

## Systemic Modulators: Shaping AAA Through Blood Lipids, Gut Microbiota, and Sex Hormones

Although AAA is often regarded as a localized vascular pathology, evidence has established that systemic factors play a significant role in its progression. Among these, dysregulated lipid metabolism, gut microbiota dysbiosis, and sex hormones are critical modulators of AAA initiation and progression.

### Dyslipidemia: a Key Systemic Driver of AAA

Dyslipidemia has long been associated with cardiovascular diseases, such as atherosclerosis and AAA. Our recent studies using genetically modified mouse models, including *Lpl*-deficient, *Apoa5*-deficient, and human *APOC3* transgenic mice, revealed that elevated triglyceride (TG) levels drive AAA development in a TG level-dependent manner [54]. Consistently, high TG levels accelerate aneurysm expansion in the PPE model [54]. Mechanistically, excess TGs impair the maturation and enzymatic activity of lysyl oxidase, a key enzyme responsible for collagen and elastin cross-linking in the ECM. Reduced lysyl oxidase activity weakens the aortic wall integrity, increasing susceptibility to dilation and rupture. Complementing these findings, genome-wide association studies by Roychowdhury et al. identified lipid metabolism as a central pathway in AAA pathogenesis [55]. Epidemiological studies have also linked elevated lipoprotein(a) levels with increased AAA risk, highlighting its potential as both a biomarker and therapeutic target [56]. In contrast, the role of hypercholesterolemia (elevated LDL-C and total cholesterol) in AAA appears to be model-dependent. In AngII-induced AAA using *Ldlr*- and *Apoe*-deficient mice, as well as C57BL/6J mice expressing a gain-of-function mutant of PCSK9, hypercholesterolemia accelerates aneurysm formation [57]. *Pcsk-9*-deficient mice with a standard chow diet, having dramatically decreased TC and HDL-C levels, displayed a significant decrease in expansion of the abdominal aortic diameter in PPE-induced AAA [55], indicating PCSK9 may have both a systemic (lipid-driven) and local (vascular inflammatory) contributor to aneurysm pathogenesis. However, Mulorz et al. reported that hypercholesterolemia does not significantly affect aneurysm progression in PPE-induced AAA, despite increased lipid deposition within the aneurysmal wall [58].

### Gut Microbiota: a Metabolic and Immune Modulator in AAA

The gut microbiota has been recognized for its influence on host immunity and cardiovascular health [59]. Recent findings suggest a specific role for microbiota in the development of AAA. Tian et al. [60] revealed significant alterations in both composition and metabolic profiles of the gut microbiota in AAA patients. Importantly, fecal microbiota transplantation from AAA patients into *ApoE*-deficient mice resulted in increased neutrophil infiltration and NET formation, thereby accelerating AAA progression [60]. This provides direct evidence that AAA-associated dysbiosis can propagate aortic inflammation in susceptible hosts. Complementary work by Shinohara et al. documented that gut microbiota facilitates the migration of splenic monocytes to the aortic wall [61]. Oral, but not intraperitoneal, administration of antibiotics reduced splenic monocyte abundance and suppressed AngII-induced monocyte mobilization into the aorta. In contrast, intraperitoneal antibiotic delivery failed to exert similar effects [61]. Another notable contributor is trimethylamine N-oxide (TMAO), a pro-inflammatory metabolite derived from gut microbiota. Elevated circulating TMAO levels have been consistently associated with both the incidence and progression of AAA across two independent patient cohorts [62].TMAO exacerbates AAA by promoting endoplasmic reticulum stress, while its inhibition significantly attenuates AAA formation and progression in animal models [62]. Despite these promising insights, considerable heterogeneity may arise from differences in diet, experimental models, host genetics, and microbial profiling approaches, all of which can profoundly influence both the composition and function of the gut microbiota. As such, rigorous validation in well-controlled settings and across diverse populations is necessary to clarify the causal role of gut microbiota and to harness its therapeutic potential.

### Sex Hormones: Critical Regulators of Sexual Dimorphism in AAA

Epidemiological studies consistently suggest that AAA is significantly more prevalent in men than in women, whereas, when present, aneurysms in women tend to grow more rapidly and are associated with worse clinical outcomes [63]. Sex hormone signaling has emerged as a critical factor underlying these sex differences in AAA pathogenesis, and recent mechanistic studies have provided important insights. Mu et al. reported that androgens exacerbate aneurysm formation by suppressing the expression of programmed cell death protein-1 in T cells [64]. And IL-6 is partially involved in the mechanism of androgen-mediated aortic aneurysm. In smoking-related AAA, nicotine exposure significantly increased MMP2 expression in the abdominal aorta of male mice, thereby amplifying AngII-induced aneurysm formation in a sex-specific manner [65]. Furthermore, work by Okuyama and colleagues indicated that androgens, rather than estrogens, downregulate LOX activity in the aortic wall, contributing to the observed sexual dimorphism in AAA formation [66]. Notably, of clinical relevance, although male sex is one of the strongest risk factors for AAA development, female patients exhibit paradoxically poorer clinical outcomes. Pouncey et al. reported that women had a 25% lower survival-to-discharge rate following aortic surgery compared with men [67]. In line with this, a multicenter retrospective cohort study demonstrated higher perioperative and long-term mortality in women, which may be attributable to older age at presentation and a higher prevalence of chronic kidney disease [68]. Further investigation is needed to improve risk stratification, guide personalized interventions, and optimize surgical outcomes in both sexes.

## Therapeutic Implications and Future Directions

Translating experimental findings in AAA into clinical applications remains a significant challenge due to limitations in animal models and the absence of a definitive causative factor. Nevertheless, the rapid expansion of mechanistic insights into AAA has opened promising avenues for therapeutic innovation (Table 1).

**Table 1:** Potential therapeutic strategies in AAA

Target	Application	Mechanism	References
Programmed cell death in VSMCs	Small-molecule inhibitors; ApoA1-mimetic synthetic HDL; Cryptotanshinone (from Salvia miltiorrhiza)	Inhibit necroptosis; reduce apoptosis; preserve mitochondrial homeostasis; suppress AAA progression	[69–71]
Neutrophil extracellular traps	Intrinsically anti-inflammatory nanoparticles	Suppress NET formation, reduce AAA progression	[72]
VSMC–immune cell crosstalk	Therapy targeting ligand-receptor interactions	Modulate intercellular signaling between VSMCs and immune cells to prevent disease progression	[73, 74]
Intraluminal thrombus	Antiplatelet / antithrombotic agents	Reduce proteolytic damage but may compromise wall stability; clinical benefit remains uncertain	[43–45, 75]
MSC-derived exosomes or EVs	Stem cell therapy; exosome nanomotors	Targeted delivery of therapeutic exosomes to AAA lesions; protect against VSMC senescence, DNA damage, and oxidative defects	[76, 77, 80]
Gut microbiota	TMAO inhibition; probiotic supplementation	Reduce harmful microbial metabolites; enhance protective effects of microbiota	[62, 81]
Lipid metabolism	Antisense oligonucleotides targeting Angptl3; statins	Lower lipoproteins, reduce inflammation, suppress AAA growth	[54, 82]

### Targeting VSMC Death and Mitochondrial Dysfunction

Developing targeted inhibitors of programmed cell death in VSMCs could improve AAA outcomes. Ros et al. identified key targets that inhibit the function of necroptosis proteins and developed small-molecule inhibitors that significantly improved AAA induced by necrotic apoptosis in mice [69]. Mitochondrial dysfunction is a key driver of VSMC death. A synthetic high-density lipoprotein composed of ApoA1 mimetic peptide and phospholipids preserves mitochondrial homeostasis and suppresses AAA progression, highlighting its therapeutic potential [70]. Nature also holds answers for AAA treatment. Cryptotanshinone, a bioactive compound extracted from Salvia miltiorrhiza, shows potential as an AAA therapeutic by targeting the Keap1-Nrf2-GSDMD-pyroptosis axis in VSMC [71]. Further research is needed to validate the feasibility of applying these strategies to human diseases.

### Immune Cell Modulation and Humanized Models

In addition to strategies aimed at maintaining the health VSMC phenotype, specific modulation of immune cells represents a promising therapeutic avenue in AAA. Hu and colleagues developed intrinsically anti-inflammatory nanoparticles engineered via nanoprecipitation, which effectively inhibited NET formation and suppressed AAA progression in a CaCl_2_-induced model, providing insights for precision therapy [72]. Notably, Cai et al. established a novel humanized AAA model by orthotopically transplanting remnant human internal mammary arteries into the immunodeficient NOD SCID Gamma (NSG) mice [29], facilitating the study of human monocyte function in a near-human aortic environment. These tools enable exploration of human-specific immune mechanisms in AAA, overcoming some limitations of traditional murine models.

### Intercellular Crosstalk as a Therapeutic Target

Targeting intercellular communication is an emerging, promising strategy for the prevention and treatment of AAA. While structural cells, such as VSMC and various immune cell subsets, each perform distinct biological roles within the aneurysmal aorta, growing evidence suggests that the crosstalk, cooperation, or antagonism between these cell types is a critical aspect of disease pathogenesis. Recent ligand-receptor interaction analyses have revealed significant upregulation of signaling pathways in AAA tissue. For example, interactions between myeloid cells and VSMCs include IL34/CSFR, CCL2/CCR2, JAG/Notch2, TGFB1/TGFB1R, and CXCL12/CXCR4, while interactions between T cells and VSMCs include FGF2/CD44, LTB/LTBR, FAM3c/LAMP1, and TNFSF12/TNFRSF25 [73]. Spatial transcriptomic analyses of the Ang II-induced AAA murine model have identified direct spatial proximity between VSMCs and GPNMB-expressing macrophages within the aortic wall, providing compelling evidence for physical and functional intercellular contact [74]. Elucidating the molecular mechanisms and functional consequences of these interactions may uncover novel therapeutic targets, enabling disruption of pathogenic cell communication networks within the aneurysmal environment.

### Antithrombotic Therapy: Balancing Risks and Benefits in AAA

ILT is another treatment option; however, whether antithrombotic therapy confers clinical benefit in AAA remains controversial. Preclinical studies have revealed that antiplatelet therapies can reduce aneurysm burden, but clinical translation has been inconsistent. A recent randomized controlled clinical trial evaluating the P2Y12 receptor antagonist ticagrelor in patients with small AAAs found no significant effect on aneurysm growth, raising questions about the efficacy of systemic antiplatelet therapy in AAA [75]. One possible explanation lies in ILT’s dual role. While antithrombotic therapy may limit platelet-driven inflammation and mitigate proteolytic damage, it could simultaneously compromise the mechanical stability of the aneurysm wall. Given this complexity, treatment decisions should be individualized, weighing the potential anti-inflammatory benefits of thrombus modulation against the risk of aneurysm destabilization.

### Stem Cell-Derived Extracellular Vesicles and Nanotechnology

Stem cell-based therapies for AAA primarily exert their protective effects through paracrine mechanisms mediated by EVs and exosomes, which act on target cells within the aortic wall to suppress aneurysm formation and growth. Exosomes derived from healthy donor mesenchymal stem cells (MSCs) carrying miR-19b-3p protect against AngII-induced AAA and VSMC senescence by modulating the MST4/ERK/Drp1 pathway [76]. Similarly, MSC-derived EVs enriched in nicotinamide phosphoribosyltransferase have been shown to attenuate cellular DNA damage and defects in oxidative phosphorylation [77]. Earlier strategies of stem cell therapy in AAA involved systemic delivery via intravenous injection or local administration into the aortic lumen or adventitia [78, 79]. To overcome these limitations, nanotechnology-based solutions have been developed. A notable example is the work by Wang et al., who engineered magnetic resonance imaging-traceable exosome nanomotors that combined magnetic field navigation with catalase-driven chemotaxis, thereby profoundly enhancing the targeted delivery of MSC-derived exosomes to AAA lesions [80].

### Targeting Systemic Modulators

The influence of the gut microbiota on AAA primarily stems from imbalances in microbial composition and metabolites, prompting therapeutic strategies that aim to eliminate harmful factors and enhance beneficial ones. Inhibition of TMAO production significantly reduces aneurysm size and severity in murine models, suggesting it as a promising metabolic target for AAA intervention [62]. However, given the complexity and inter-individual variability of the gut microbiome, further studies are still required to optimize strategies that maximize benefit while minimizing adverse effects. While not yet extensively studied in AAA, oral probiotic supplementation has shown favorable efficacy and tolerability in kidney injury and renal failure [81]. Given the overlapping mechanisms of oxidative stress, inflammation, and endothelial dysfunction in both AAA and renal pathology, probiotics may represent a low-risk, adjunctive therapy to modulate systemic inflammation and microbial homeostasis in AAA.

Moreover, as the role of lipid metabolism in AAA pathogenesis becomes increasingly apparent, controlling lipid levels and related metabolic factors may become critical preventive and therapeutic approaches, particularly considering the shared or overlapping mechanisms between AAA and atherosclerosis [4]. Importantly, therapeutic silencing of Angptl3 with antisense oligonucleotides significantly attenuated aneurysm formation in human *APOC3* transgenic mice and *Apoe*-deficient mice, underscoring the translational potential of targeting TG-rich lipoproteins in AAA prevention and treatment [54]. Statins, long established for lipid-lowering, have also shown promise in the treatment of AAA. A five-year prospective cohort study revealed that high-dose statin therapy was associated with a slower AAA growth rate in men with small aneurysms, supporting their therapeutic potential in AAA [82]. Further mechanistic studies are warranted to fully understand how statins and other lipid-modifying agents influence aneurysmal biology and whether their efficacy differs across AAA subtypes or patient populations.

### Biomarker Discovery and Imaging Advances

Currently, the maximum aortic diameter remains the gold standard for both diagnosing and assessing the prognosis of AAA [83]. However, this metric alone lacks sensitivity and specificity in predicting rupture risk or early disease detection. Emerging studies offer promising candidates across both molecular and imaging-based modalities (Table 2). ILT, frequently present in the aneurysmal segment, is gaining recognition as a reservoir of biomarker-relevant molecules. Soluble glycoprotein VI (GPVI), a platelet-specific activation marker, has shown superior correlation with aneurysm growth rate compared to traditional markers such as D-dimer [44]. Circulating monocytes may also serve as potential biomarkers, given their phenotypic and activation state changes during AAA development. In addition, some case-control studies have identified significant associations between blood neutrophil and lymphocyte levels and the presence of AAA [84]. To fully realize the clinical potential of these markers, integrating hematological features into multifactorial models using machine learning or artificial intelligence may improve their diagnostic and prognostic accuracy.

**Table 2:** Potential biomarkers in AAA

Target	Application	Mechanism	References
ILT-derived platelet activation	Soluble glycoprotein VI (GPVI)	Diagnostic/prognostic marker; correlates with AAA growth rate	[44]
Circulating immune cells	Biomarker of immune activation; risk stratification	Reflect phenotypic/activation changes during AAA development; correlate with AAA presence and progression	[24, 84]
CXCR4	Molecular imaging probe (Fe₃O₄-anti-CXCR4-PE)	Detects immune/inflamed vascular cells in vivo; imaging biomarker of inflammation	[85]
PVAT attenuation (FAI)	CT angiography biomarker	Quantifies local vascular inflammation; correlates with AAA progression	[86]
PVAT radiomic features	Prognostic marker after EVAR	Higher surface area-to-volume ratio and heterogeneity predict aneurysm expansion	[87]

Advances in imaging technologies have further improved the capacity for early AAA detection and prognosis assessment. For example, the chemokine receptor CXCR4 has emerged as a promising molecular marker of immune cell recruitment in vascular disease. Cao et al. developed Fe₃O₄-anti-CXCR4-PE probes that enabled the detection of immune cells and inflamed vascular cells in murine AAA models using both magnetic particle and fluorescence imaging, offering real-time visualization of systemic inflammation [85]. While promising, further studies are needed to confirm whether CXCR4 imaging reflects AAA pathology reliably across diverse clinical contexts.

PVAT-based imaging biomarkers also hold considerable potential. The fat attenuation index, originally developed and validated in coronary artery disease to quantify local vascular inflammation [49], has shown preliminary utility in AAA. Retrospective studies have reported that elevated PVAT attenuation values on computed tomography angiography independently correlate with AAA progression [86]. Moreover, radiomic analysis has revealed that textural and structural features of PVAT, such as a higher surface area-to-volume ratio and heterogeneity, are associated with post-EVAR aneurysm expansion, making PVAT imaging a promising prognostic tool [87].

## Conclusion

Abdominal aortic aneurysm remains a life-threatening vascular disease with limited therapeutic options beyond surgical intervention. Recent advances in single-cell and spatial transcriptomics, together with mechanistic studies in both human tissues and animal models, have significantly deepened our understanding of the disease’s multifactorial nature. These investigations have revealed a complex network of interactions involving lesion-associated cells (VSMC, EC, and immune populations), local vascular microenvironment (ILT and PVAT), and systemic modulators (lipid metabolism, gut microbiome, and sex hormones), all of which collectively drive aneurysm initiation and progression. Importantly, this expanding body of knowledge has unveiled multiple therapeutic entry points. While ongoing mechanistic exploration remains essential, future efforts must increasingly emphasize clinical translation. Key priorities include the development of robust biomarkers for early detection and risk stratification, refinement of non-invasive imaging technologies, and validation of findings in humanized or patient-representative models, paving the way for a new era of mechanism-guided, personalized therapies for AAA.

## Data Availability

No datasets were generated or analysed during the current study.
